# A peripheral giant cell granuloma with extensive osseous metaplasia or a hybrid peripheral giant cell granuloma-peripheral ossifying fibroma: a case report

**DOI:** 10.1186/1752-1947-9-14

**Published:** 2015-02-04

**Authors:** Ezinne I Ogbureke, Nadarajah Vigneswaran, Matthew Seals, Gary Frey, Cleverick D Johnson, Kalu UE Ogbureke

**Affiliations:** 9 Department of General Practice and Dental Public Health, The University of Texas School of Dentistry, Houston, TX USA; 10 Department of Diagnostics and Biomedical Sciences, The University of Texas School of Dentistry, Houston, TX USA

**Keywords:** Collision/hybrid lesion, Dental implants, Peripheral giant cell granuloma, Peripheral giant cell granuloma with osseous metaplasia, Peripheral ossifying fibroma

## Abstract

**Introduction:**

Peripheral giant cell granuloma and peripheral ossifying fibroma are clinicopathologically distinct gingival lesions. Both are included in clinical differential diagnoses of common benign and reactive gingival epulides in humans. It is often impossible to make a clinical distinction between the two entities, thereby making definitive diagnosis dependent on histopathologic features. While our search of the English literature revealed several reports of peripheral giant cell granuloma with ‘bone formation’, we were unable to identify any reports of hybrid peripheral ossifying fibroma-peripheral giant cell granulomas.

**Case presentation:**

We report a case of a 44-year-old Caucasian man presenting with a three-month history of swelling of his right posterior mandible, related to an area of previous dental implant restoration. A clinical examination revealed modest extraoral facial swelling of his right posterior mandible, while an intraoral examination showed a 45×25×15mm sessile, lobular soft tissue mass of the right posterior mandibular gingiva. The mucosal covering of the lesion exhibited focal surface ulceration. A panoramic radiograph showed two implants at the vicinity of the lesion with no other significant findings. An excisional biopsy of the lesion followed by histopathologic examination of the biopsy specimen revealed salient and distinctive features of peripheral giant cell granuloma and of peripheral ossifying fibroma, estimated at near equal proportions. This raises the possibility of a hybrid odontogenic lesion.

**Conclusion:**

The presentation of this lesion, with areas of peripheral giant cell granuloma along with a distinct area of extensive osseous formation and stroma reminiscent of a peripheral ossifying fibroma, justifies consideration of this as a possible hybrid lesion. Although the biologic behavior of a combined lesion is not anticipated to deviate significantly from that of either of the single entities, this case resurrects an enduring debate as to whether peripheral giant cell granuloma and peripheral ossifying fibroma are simply parts of a disease spectrum, or whether some of these lesions represent true hybrid lesions. It is therefore recommended that more cases with histopathologic features similar to the lesion in our case be reported in the literature to further elucidate the histogenesis of these lesions.

## Introduction

Peripheral ossifying fibroma (POsF) was first reported and described by Shepherd as ‘alveolar exostosis’ in 1844 [1]. Because this lesion presents with a spectrum of histomorphologic features, several subsequent reports have characterized it variously as peripheral fibroma with calcification, calcifying fibroblastic granuloma, ossifying fibroid epulis, peripheral cemento-ossifying fibroma, and calcifying fibroma [2, 3]. The terminology has since stabilized, with this entity now referred to as POsF, peripheral cementifying fibroma, or peripheral cement-ossifying fibroma, depending on whether bone, cementum, or proportions of each are present on microscopy [3–7]. Similarly, peripheral giant cell granuloma (PGCG) was first reported as fungus flesh in 1848 [4], then reported as giant cell reparative granuloma by Jaffe in 1953 [8]. Subsequent reports also featured a constellation of terminology such as osteoclastoma, giant cell epulis, and myeloid epulis [3]. PGCG is now the preferred terminology.

POsF and PGCG are site-specific lesions arising exclusively from the periodontal ligament. This makes both, by definition, lesions of the gingiva or alveolar ridge [9, 10]. Their histogenesis is distinct from that of their respective intraosseous (central) namesakes, central ossifying fibroma (COF) and central giant cell granulomas (CGCG), which are intra-bony benign neoplasms of the jawbone [10]. Thus, POsF and PGCG are regarded as reactive lesions of gingiva, often presenting as painless, lobular, and ulcerated masses that are clinically indistinguishable from one another.

Cases of both POsF and of PGCG are occasionally seen with isolated foci of the diagnostic histopathologic feature of the other, but the preferred diagnosis of one over the other is usually based on the predominant morphologic features present in any specific case. Thus, cases with predominantly stromal cells with numerous osteoclast-like multinucleated giant cells with only focal areas of calcified bone and/or cementum deposits are diagnosed as PGCG. Those with a stroma comprising aggregates of primitive oval and bipolar mesenchymal cells, where a trabecular of woven and lamella bone with cementum-like deposit often dominates, receive a POsF diagnosis [11]. Archival papers by Dayan *et al*. [12] and similar reports by Katsikeris *et al*. [13] highlighted the overlaps in the histopathologic features of PGCG and POsF. Here, we report on a case of PGCG with a distinct region with an extensive osseous component and other stromal features of POsF that may qualify this lesion as a true case of hybrid PGCG-POsF. We also discuss the pathogenesis of this lesion.

## Case presentation

A 44-year-old Caucasian man presented to our Urgent Care clinic with a complaint of swelling in his right posterior mandibular molar teeth area that started about three months previously. There was no associated pain except for occasional interference of the swelling with occlusion and mastication. His vital signs were within normal ranges; his medical history included a family history of diabetes, cardiovascular disease, and cancers. His previous dental treatment included two right posterior quadrant dental implants, proximate to the lesion area, placed three months prior to presentation.

**Figure 1:**
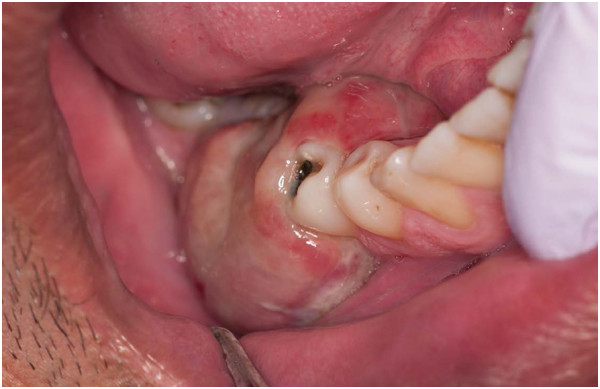
**Clinical photograph of intraoral gingival soft tissue swelling in the area of the right lower mandibular molar teeth.**

**Figure 2:**
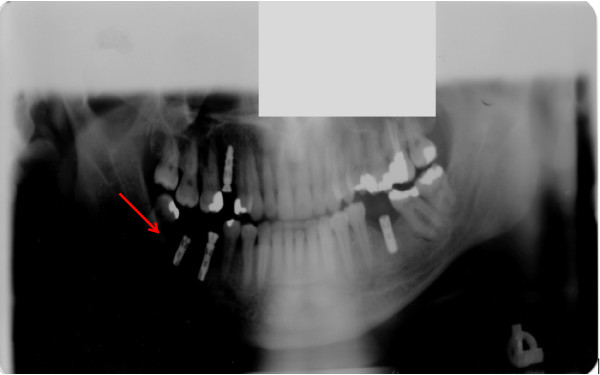
**Panoramic radiograph of patient’s jaws.** Shows no evidence of intra-bony pathology in association with the soft tissue lesion (arrow).

On clinical examination, he had slight extraoral facial swelling of his right posterior mandible, but his regional lymph nodes were not palpable. An intraoral examination showed a 45×25×15mm sessile, lobular soft tissue mass of his right posterior mandibular gingiva related to his first and second premolars (Figure 1). The mucosal covering of the lesion exhibited surface tan, red, and bluish areas with a focal area of ulceration. A panoramic radiograph of his jaws revealed two implants at his right posterior mandible in the vicinity of the lesion (Figure 2) with no other significant findings. These clinical and radiographic findings indicated a benign lesion, and the following differential diagnoses were generated: pyogenic granuloma, POsF, peripheral odontogenic fibroma, focal fibroepithelial hyperplasia, and PGCG. Malignant entities such as squamous cell carcinoma, other primary malignant lesions, and metastatic lesions, although thought to be unlikely, were also considered. Complete excision of the lesion was performed, and the entire specimen submitted for histopathologic examination.

**Figure 3:**
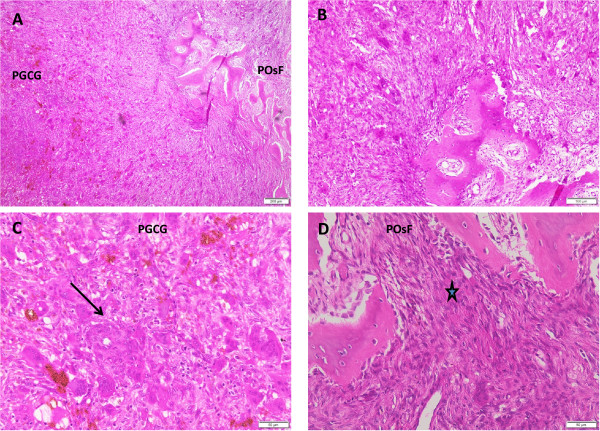
**Histopathologic micrograph of combined peripheral ossifying fibroma and peripheral giant cell granuloma.** Hematoxylin and eosin-stained sections showing PGCG and POsF areas of lesion at **(A)** 10× magnification and **(B)** 20× magnification. **(C)** Same staining predominantly showing the PGCG area and prominent aggregates of multinucleated giant cell reaction (*) at 40× magnification. **(D)** Same staining showing a predominantly POsF area (arrow) within the hybrid lesion at 40× magnification. POsF, peripheral ossifying fibroma; PGCG, peripheral giant cell granuloma.

Microscopic examination of the hematoxylin and eosin-stained sections of the specimen revealed a nodular soft tissue specimen consisting of an ulcerated benign cellular mesenchymal tissue proliferation supporting elaborate trabecular bone formation, and occasional cementum-like calcified deposits (Figure 3A,B,D). This interfaced with stroma supporting aggregates of benign multinucleated giant cells with hemorrhagic and hemosiderin deposits (Figure 3A-3C). This framework was covered by discontinuous parakeratinized surface stratified squamous epithelium maintaining the usual pattern of cellular maturation, which rested on an intact basal cell layer with elongated rete ridges. Where the surface epithelium was discontinuous it was covered by a fibrinous layer supporting neutrophils and extravasated erythrocytes.

## Discussion

Various combinations of so-called hybrid odontogenic lesions have been reported [14]. These include reports of several series on combined COF with CGCG [14–16]. For example, Allen *et al*. [15] reported three cases of ‘epithelium-rich’ COF, with ‘unusual associated giant cell reaction’, whereas Tosios *et al*. [14] reported seven cases of hybrid central giant cell lesions (CGCLs) and COFs of the jaws. Odell *et al*. [16] gave a detailed microscopic description of eight patients with ‘hybrid central giant cell granuloma and central odontogenic fibroma-like’ lesions. There are also reports of CGCL with ameloblastoma, and several non-odontogenic fibro-osseous lesions [17, 18]. Although the case series reported by Dayan *et al*. [12] and Katsikeris *et al*. [13] highlighted the formation of mineralized tissues in PGCG, they did not find cementum-like calcifications in the mineralized matrix in their respective series. To the best of our knowledge, our case is the first reported in which the PGCG area is distinct from the mineralized area and present in near equal proportion, and the mineralized stroma contains occasional cementum-like matrix.

POsFs are reactive lesions distinct from COFs, which are true intraosseous neoplasms. Similarly, PGCGs are reactive lesions that do not represent the intraosseous counterpart of CGCG. In both pairings the peripheral lesions present as indistinguishable histopathologic look-alikes, as do the corresponding central lesions. Because of the contiguity of the gingival and alveolar apparatus to the alveolar bone, mandible, and maxilla, the presentation of these entities occasionally raises the possibility of either an intra-bony (mandible or maxilla) lesion that eroded to the surface and into the overlying gingival soft tissue, or an originally soft tissue lesion that burrowed its way into bone [11]. In our case there was no radiographic evidence of alveolar bone involvement, thereby establishing the diagnosis of a soft tissue entity.

Dayan *et al*. [12] emphasized the absence of cementum deposits or matrix in PGCG with extensive osseous elements as a basis for characterizing these lesions as PGCG with osseous formation or metaplasia, instead of as true hybrid PGCG-POsF lesions. While we agree that cases of osseous metaplasia in PGCG may be common, we suggest that the absence of cementum-like elements alone is not sufficient to reject all such lesions as true PGCG-POsF hybrid lesions. This is because cases of POsF lacking distinct cementum-like deposits are not uncommon. Thus a diagnosis of POsF is based on the presence of distinct and characteristic stroma, comprising intertwining bundles of collagen admixed with haphazardly arranged fibroblast-like cells, in association with bone and/or cementum-like material. Our case showed a distinct area satisfying the histopathologic features of POsF (Figure 3D). The POsF area was unlikely to represent ‘exuberant callus’ because, although common in long bones, this is almost never seen at the surface of jaw bones [11]. Furthermore, there was a noticeable paucity of red cell extravasation in the stroma of the POsF portion of the lesion compared with the PGCG portion (Figure 3C), which showed characteristic exuberance of red cell extravasation accompanied by hemosiderin deposits [11].

Both POsF and PGCG are reactive inflammatory hyperplasias arising from the pluripotent cells of the periodontal ligaments [11]. It is therefore conceivable that both entities represent points on a spectrum of the same reactive disease process. While the implant treatment in the vicinity of the lesion may have constituted a persistent inflammatory trigger or irritant, we suggest the possibility that the POsF and PGCG arose *de novo*. Cases of PGCG associated with dental implant therapies have been reported [19–21]. In most of the cases the implant and restoration materials were composed of coronal prosthesis and were associated with poor oral hygiene [20, 21].

## Conclusion

We report a case of PGCG with a distinct area of extensive osseous formation and stromal area, reminiscent of that characteristic of POsF. The combined classic histopathologic features of both lesions represented in similar proportions justify the diagnosis of a combined PGCG-POsF. The biologic behavior of the combined lesion is not anticipated to deviate significantly from that of either of the lesions in their commonly single entity presentations. We recommend that more cases with histopathologic features similar to our case be reported in the literature to further elucidate the histogenesis of these lesions.

## Consent

Written informed consent was obtained from the patient for publication of this case report and any accompanying images. A copy of the written consent is available for review by the Editor-in-Chief of this journal.

## Abbreviations

CGCL: central giant cell lesion
COF: central ossifying fibroma
PGCG: peripheral giant cell granuloma
POsF: peripheral ossifying fibroma.
